# A Review of Clinical Trials That Contributed to Chronic Obstructive Pulmonary Disease Treatment Protocols

**DOI:** 10.7759/cureus.14618

**Published:** 2021-04-21

**Authors:** Jude ElSaygh, Anas Zaher, Pratiksha Nathani, Mohamed Omballi

**Affiliations:** 1 Internal Medicine, University of Debrecen, Debrecen, HUN; 2 Internal Medicine, Maharashtra University of Health Sciences, Latur, IND; 3 Pulmonary and Critical Care Medicine, The University of Toledo, Toledo, USA

**Keywords:** copd, torch, uplift, poet, wisdom, rct, obstructive lung disease, tiotropium, salmeterol

## Abstract

Chronic obstructive pulmonary disease (COPD) has remained a leading cause of death worldwide and is expected to increase its burden on the healthcare system in the coming future. Numerous clinical trials have been conducted over the years and as a result, many drugs became a part of the treatment protocols of COPD. Currently, there are also several drugs under development. This review will help future researchers to grasp salient features of previous studies and use them in their future trials in order to reduce the morbidity and mortality of COPD. Randomized control trials provide strong evidence for any hypothesis in a research study. This review focuses on major COPD trials in the last two decades including TORCH, UPLIFT, POET, WISDOM, and TIOSPIR. It showcases the main clinical question, primary outcome, and result of these five trials.

## Introduction and background

Chronic obstructive pulmonary disease (COPD) is a progressive irreversible inflammation disease affecting the airways, alveoli, and microvasculature. It is often preventable and treatable. The global prevalence of COPD was measured at around 174 million in the World Burden of Disease Survey 2015. In 2015, COPD came third among the world's age standard mortality rates for both sexes with about 32 million deaths [1]. Moreover, COPD's financial burden accounted for almost $50 billion in government spending in 2010 [2]. 

Pharmacologic treatments are used to relieve symptoms, reduce the incidence and severity of exacerbations, and enhance exercise tolerance and overall health [3]. Classically, there are two groups of drugs used in an exacerbation. The first group is the bronchodilators which include: short-acting beta 2 agonists (SABA), long-acting beta 2 agonists (LABA), short-acting muscarinic antagonists (SAMA), and long-acting muscarinic antagonists (LAMA). The second group is the anti-inflammatory drugs which include: inhaled corticosteroids (ICS), phosphodiesterase-4 inhibitors (PDE4i), macrolides (azithromycin), and mucoactive agents with antioxidant effects [4,5]. There are newer classes of drugs known as immunomodulators that aim to target the pathophysiology of COPD rather than focusing on treating the symptoms [6]. This review will highlight the major clinical trials that were used to establish treatment protocols in COPD.

## Review

**Figure 1:**
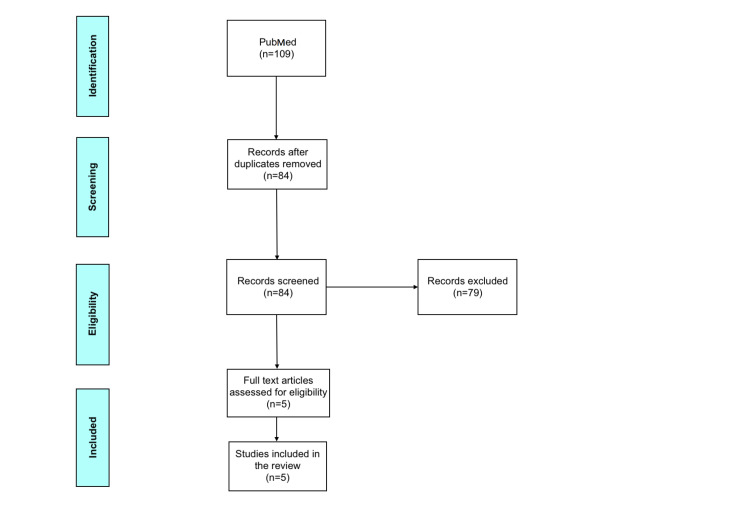
Flow diagram to illustrate selection criteria of chronic obstructive pulmonary disease trials

A review of studies from 2000 to 2020 involving therapeutic interventions on patients with COPD was conducted. The goal of this article is to present major interventional clinical trials conducted in the last two decades. This will help connect the dots and play a role in creating up-to-date guidelines for COPD management. A literature search was done on PubMed using keywords such as COPD, Tiotropium, Salmeterol, LABA, LAMA, Bronchodilators, Inhalational Corticosteroids, Glucocorticoids, Fluticasone Propionate, Exacerbations, Eosinophil Count, protocol, GOLD as free texts or as MeSH terms. This led to the retrieval of 109 abstracts. The criteria for exclusion were: (1) observational studies, (2) studies that did not include the drugs in the GOLD 2020 guidelines, (3) studies with participants with lung disorders other than COPD, and (4) studies with less than 2000 participants. After applying the exclusion criteria, five full-text articles were selected (Figure 1).

Towards a revolution in COPD health (TORCH) survival study (September 2000 - November 2005)

Prior to the year 2000, LABA and ICS were used in the treatment of COPD. They were effective in reducing exacerbations. However, whether or not they had any survival benefit was not studied up to that point in time. The main objective of this trial was to study the percentage reduction in mortality in the salmeterol + fluticasone propionate, salmeterol alone, fluticasone propionate alone, or placebo groups. The clinical question that this trial was trying to answer is if the combination of a LABA and ICS is more effective in reducing mortality than either agent alone. The duration of the trial was five years. It was a randomized and double-blinded trial. The participants were required to be 40- to 80-year-old patients with COPD, have forced expiratory volume in one second (FEV1) <60% of predicted normal and a baseline (pre-bronchodilator) ratio of FEV1 to forced vital capacity (FVC) of <70%. Only current or former smokers with at least 10-pack-year were included.

The four arms of the study were: salmeterol + fluticasone propionate, salmeterol alone, fluticasone propionate alone, placebo. The primary outcome measured was the death from any cause and the comparison of this parameter between the combination regimen and placebo group. The proportions of deaths from any cause at three years were 12.6% in the combination therapy group, 15.2% in the placebo group, 13.5% in the salmeterol group, and 16.0% in the fluticasone group. However, the reduction in death from all causes among patients with COPD in the combination-therapy group did not reach the predetermined level of statistical significance. There were significant benefits in all other outcomes among these patients. A reduction of 25% in the annual rate of COPD exacerbations in the combination group compared to the placebo group was found. Averaged over three years, the health status (a reduction of 3.1 units in the score for the St. George's Respiratory Questionnaire) and spirometric measurements (an increase in FEV1 of 0.092 liters) in the combination therapy group were significantly better than in the groups receiving placebo, salmeterol alone, or fluticasone propionate alone.

There were two potential explanations of the results provided by the TORCH investigators. The first one: there is no effect on the combination of fluticasone propionate and salmeterol in improving survival, and the second one: the combination of fluticasone propionate and salmeterol has survival benefits in COPD patients, but the study was underpowered to detect this [7,8].

Understanding the potential long-term impacts on function with tiotropium (UPLIFT) trial (December 2002 - February 2008)

It’s arguably one of the largest and most well-designed trials regarding COPD. The UPLIFT trial included around 6000 patients. This trial helped physicians better understand the long-term impacts of tiotropium use on lung function. The main clinical question of this study was whether tiotropium reduced the rate of decline of FEV1 in COPD patients. The duration of the trial was four years. It was also a randomized and double-blinded trial. The participants were required to be at least 40 years of age, with an FEV1 of 70% or less after bronchodilation and a ratio of FEV1 to FVC of 70% or less.

The two arms of this trial were tiotropium users and the placebo group which was permitted to use all respiratory medications except inhaled anticholinergic drugs. The primary outcome measured was the rate of decline in the mean FEV1 before and after bronchodilation beginning on day 30. After day 30, the differences between the two groups in the rate of decline in the mean FEV1 before and after bronchodilation were not significant. However, at 4 years and 30 days, tiotropium was associated with a reduction in the risks of exacerbations, related hospitalizations, and respiratory failure. St. George's Respiratory Questionnaire was improved in the tiotropium group, as compared with the placebo group, at each time point throughout the four-year period. This study concluded that Tiotropium treatment in COPD patients was linked to the reduction of COPD exacerbations over a span of four years but did not decrease FEV decline. In terms of the adverse effects of long-term use of tiotropium, the UPLIFT trial was reassuring in the safety of tiotropium. It showed lower rates of cardiac and respiratory complications compared to the placebo group [9,10].

Prevention of exacerbations with tiotropium in COPD (POET-COPD) trial (January 2008 - April 2010)

Picking a suitable and more effective bronchodilator is key in managing COPD patients. Between beta 2 agonists and anticholinergics, which is superior? Are they equal in reducing exacerbations of COPD? POET-COPD finally derived the answer to this question. In this study, the question being asked is whether tiotropium 18 μg once daily was superior to salmeterol 50 μg twice daily in reducing COPD exacerbations. The duration of the trial was one year. It was a randomized, double-blinded, double-dummy, parallel-group trial. Participants were required to be at least 40 years of age and had a smoking history of 10 pack-years or more, with a diagnosis of COPD, an FEV1 after bronchodilation of ≤70% of the predicted value, a ratio of FEV1 to FVC of ≤70%, and a documented history of at least one exacerbation leading to treatment with systemic glucocorticoids or antibiotics or hospitalization within the previous year.

The two arms of the study were patients using tiotropium and patients using salmeterol. The primary outcome measured was the time to the first exacerbation of COPD. The study found that tiotropium, compared with salmeterol, increased the time to the first exacerbation (187 days vs. 145 days), with a 17% reduction in risk (hazard ratio, 0.83; 95% confidence interval [CI], 0.77 to 0.90; P<0.001). Tiotropium use was also found to reduce the annual number of moderate or severe exacerbations of COPD compared to salmeterol. This study concluded that tiotropium is more effective than salmeterol in terms of reducing the number of COPD exacerbations and increasing the time to first exacerbation after using the drug compared to salmeterol [11,12].

Withdrawal of ICS treatment in patients with severe to very severe COPD on optimized bronchodilator therapy (WISDOM) trial (February 2009 - July 3013)

In patients with frequent exacerbations of severe COPD, inhaled glucocorticoids in combination with long-acting bronchodilators are generally recommended. In comparison to long-acting bronchodilators, the advantage of inhaled glucocorticoids was not thoroughly studied. In WISDOM, patients initially on salmeterol, tiotropium, and glucocorticoids were stripped off the glucocorticoid for three months. The clinical question that this trial was trying to answer is whether the discontinuation of ICSs is associated with an increase in the frequency of COPD exacerbations in patients with severe COPD receiving triple therapy (tiotropium, salmeterol, and fluticasone). The duration of the trial was one year. It was a double-blinded, parallel-group study. Participants were required to be at least 40 years of age and either current smokers (roughly 10 pack-years) or former smokers. They had to be diagnosed with severe or very severe COPD, described as FEV1, less than 50% of the predicted volume and less than 70% of the FVC after bronchodilation, and have a history of at least one documented exacerbation in the 12 months before screening.

The arms of the study were tiotropium inhalation, salmeterol xinafoate, and fluticasone propionate or tiotropium inhalation, salmeterol xinafoate, and fluticasone propionate replacing fluticasone propionate with placebo for three months. The primary outcome measured was the time to first moderate or severe on-treatment COPD exacerbation. At 12 months, there was no statistically significant difference between groups for the primary outcome of time to the first moderate/severe COPD exacerbation. Tapering off the inhaled corticosteroid was not associated with an increase in the rate of COPD exacerbation. However, the withdrawal group had a moderate decrease in FEV1 from the baseline compared to the fluticasone maintenance group. The conclusion of the study found that the removal of glucocorticoids in a stepwise manner from the triple therapy was non-inferior to the continuation of triple therapy in terms of COPD exacerbations. However, the decline in FEV1 in the withdrawal group is worth noting [13,14]. 

Comparison of tiotropium in the HandiHaler Versus the Respimat in COPD (TIOSPIR) study (May 2010 - May 2013)

As per the UPLIFT trial, tiotropium Handihaler was associated with lower mortality rates than placebo, whereas mortality rates with tiotropium Respimat were associated with higher mortality rates than placebo. TIOSPIR aimed to study this discrepancy further. This trial's main clinical question was whether there is a true difference between tiotropium Handihaler and tiotropium Respimat in terms of all-cause mortality and time to first COPD exacerbation. The duration of the trial was three years. It is a randomized, double-blinded, parallel-group trial. Participants were required to be 40 years or older with relatively stable airway obstruction with a post-bronchodilator FEV1 ≤ 70% of predicted normal and post-bronchodilator FEV1/FVC ≤70%, and current or ex-smokers with a smoking history of ≥10 pack years.

The arms of this study were: tiotropium 1.25 µg 2 puffs/day via soft mist inhaler, tiotropium 18 µg via Handihaler, tiotropium 2.5 µg 2 puffs/day via soft mist inhaler. The primary outcome measured was the time to all-cause mortality and the time to first COPD exacerbation. This trial found that there is no statistically significant difference between tiotropium Handihaler and tiotropium Respimat in terms of all-cause mortality and time to first COPD exacerbation. Therefore, the conclusion was that the safety profile and exacerbation efficacy of Tiotropium Respimat at a dose of 5 μg or 2.5 μg were close to that of Tiotropium HandiHaler at the 18 μg dose in COPD patients. One justification of this corrected discrepancy could be that in the Tiospir study, discontinuation rates in the three study groups were equivalent and lower than those from the UPLIFT study. A greater percentage of patients in the placebo group discontinued than in the treatment groups in the UPLIFT trial [15,16].

Additional questions answered from the five trials mentioned above

The UPLIFT Trial

A secondary analysis of the UPLIFT trial investigated the effect of tiotropium inhalation once daily in patients who did not receive other maintenance therapy at inclusion (maintenance naive subgroup). It was found that patients in whom tiotropium was started as the first maintenance drug had evidence of slowed COPD progression. Patients in the tiotropium group showed a clinically important difference in health-related quality of life. In addition, trough FEV1 was 134 mL better in patients treated with tiotropium compared to control subjects [17].

The POET Trial

Investigators found that there was a certain genetic difference in patients who benefited more from LAMA than LABA. Polymorphisms of the gene encoding the β2-adrenergic receptor (ADRB2) were among the gene targets studied. The results of this subanalysis of the POET-COPD trial showed that Arg16 homozygous patients had better exacerbation outcomes in response to salmeterol than did patients carrying the Gly16 allele, suggesting a potential differential Arg16Gly genotype effect on treatment response to LABAs [18].

The WISDOM Trial

Blood eosinophil counts were linked to the exacerbations after complete glucocorticoid withdrawal. Data suggested that counts of 4% or greater or 300 cells per μL or more might relate to deleterious effects of glucocorticoid withdrawal, which were not seen in most patients with eosinophil counts below these counts [19].

Awaited trials regarding COPD are listed in Table 1.

**Table 1:** Awaited trials regarding chronic obstructive pulmonary disease Derived from https://clinicaltrials.gov/.

Awaited trials	Expected reporting
Anti-inflammatory effects of tiotropium in patients with stable COPD (ANTIOFLAM)	December 2020
Impact of inhaled PT003 on complexity and variability of tidal breathing and oscillatory mechanics in stable COPD patient (OSCIVARI)	February 2021
Study comparing dual combination of product (budesonide and formoterol) given via two different inhalers. To see which one results in the best effect on breathing.	May 2021
RETHINC: redefining therapy in early COPD for the pulmonary trials cooperative	July 2021
Dose of corticosteroids in COPD (DOSE)	July 2021
Salmeterol/fluticasone 50/500 mcg inhalation powder via Capsair vs Seretide Diskus 500 mcg inhalation powder in patients with COPD	December 2021
Arbidol for COPD exacerbations	August 2022
Tezepelumab COPD exacerbation study (COURSE)	March 2023
Roflumilast or azithromycin to prevent COPD exacerbations (RELIANCE)	February 2023
Pivotal study to assess the efficacy, safety, and tolerability of Dupilumab in patients with moderate to severe COPD with type 2 inflammation (NOTUS)	March 2023

## Conclusions

Numerous aspects of COPD management will need further study, and inevitably more questions regarding our current guidelines will arise. A more detailed picture of the pathophysiology of chronic obstructive lung disease will help guide better management options for COPD patients. The drugs currently available and the ones yet to be developed need to be studied for reversibility of the damage. 

COPD remains a leading cause of death worldwide. This suggests that the treatment guidelines followed today are not effective in the control of the mortality and morbidity of the disease. There is still room for substantial improvement. Major clinical trials recruiting a large number of COPD patients need to be performed for an extended duration to evaluate the survival benefits in long term. The association of existing comorbidities also needs to be thoroughly studied as a contributing factor to the rise of morbidity and mortality in COPD.
